# Gendered health, economic, social and safety impact of COVID-19 on adolescents and young adults in Nairobi, Kenya

**DOI:** 10.1371/journal.pone.0259583

**Published:** 2021-11-09

**Authors:** Michele R. Decker, Shannon N. Wood, Mary Thiongo, Meagan E. Byrne, Bianca Devoto, Rosemary Morgan, Kristin Bevilacqua, Anaise Williams, H. Colleen Stuart, Grace Wamue- Ngare, Lori Heise, Nancy Glass, Philip Anglewicz, Elizabeth Gummerson, Peter Gichangi

**Affiliations:** 1 Department of Population, Family and Reproductive Health, Johns Hopkins Bloomberg School of Public Health, Baltimore, MD, United States of America; 2 Department of Population, Family and Reproductive Health, Bill & Melinda Gates Institute for Population and Reproductive Health, Johns Hopkins Bloomberg School of Public Health, Baltimore, MD, United States of America; 3 International Centre for Reproductive Health-Kenya, Nairobi, Kenya; 4 Department of International Health, Johns Hopkins Bloomberg School of Public Health, Baltimore, MD, United States of America; 5 Johns Hopkins Carey Business School, Baltimore, MD, United States of America; 6 Department of Sociology, Gender and Development Studies, Kenyatta University, Nairobi, Kenya; 7 Women’s Economic Empowerment Hub, Kenyatta University, Nairobi, Kenya; 8 Johns Hopkins School of Nursing, Baltimore, MD, United States of America; 9 Center for Global Health, Johns Hopkins University, Baltimore, MD, United States of America; 10 Technical University of Mombasa, Mombasa, Kenya; 11 Department of Public Health and Primary Care, Faculty of Medicine and Health Sciences, Ghent University, Ghent, Belgium; University of Connecticut, UNITED STATES

## Abstract

**Background:**

Infectious disease outbreaks like COVID-19 and their mitigation measures can exacerbate underlying gender disparities, particularly among adolescents and young adults in densely populated urban settings.

**Methods:**

An existing cohort of youth ages 16–26 in Nairobi, Kenya completed a phone-based survey in August-October 2020 (n = 1217), supplemented by virtual focus group discussions and interviews with youth and stakeholders, to examine economic, health, social, and safety experiences during COVID-19, and gender disparities therein.

**Results:**

COVID-19 risk perception was high with a gender differential favoring young women (95.5% vs. 84.2%; p<0.001); youth described mixed concern and challenges to prevention. During COVID-19, gender symmetry was observed in constrained access to contraception among contraceptive users (40.4% men; 34.6% women) and depressive symptoms (21.8% men; 24.3% women). Gender disparities rendered young women disproportionately unable to meet basic economic needs (adjusted odds ratio [aOR] = 1.21; p<0.05) and in need of healthcare during the pandemic (aOR = 1.59; p<0.001). At a bivariate level, women had lower full decisional control to leave the house (40.0% vs. 53.2%) and less consistent access to safe, private internet (26.1% vs. 40.2%), while men disproportionately experienced police interactions (60.1%, 55.2% of which included extortion). Gender-specific concerns for women included menstrual hygiene access challenges (52.0%), increased reliance on transactional partnerships, and gender-based violence, with 17.3% reporting past-year partner violence and 3.0% non-partner sexual violence. Qualitative results contextualize the mental health impact of economic disruption and isolation, and, among young women, privacy constraints.

**Implications:**

Youth and young adults face gendered impacts of COVID-19, reflecting both underlying disparities and the pandemic’s economic and social shock. Economic, health and technology-based supports must ensure equitable access for young women. Gender-responsive recovery efforts are necessary and must address the unique needs of youth.

## Introduction

Gender is a potent social determinant of health [1, 2]. In global health emergencies, endemic gender disparities can be amplified. Crisis and its aftermath can threaten women’s economic and social standing by reducing autonomy and participation in paid labor, while increasing economic dependence, domestic work, and risk of gender-based violence [3–6]. The COVID-19 pandemic correspondingly presents a disproportionate social and economic risk to women and girls [3], possibly reversing tenuous advances in gender equality [4, 5].

After Kenya’s first case of COVID-19 was identified on March 13, 2020, business and school closures were swiftly implemented, along with social restrictions including a local curfew in Nairobi [7]. Densely populated urban Nairobi confers underlying socio-economic and gender inequities that risk disparate COVID-19 impact and present challenges in maintaining preventive measures, such as restrictions on movement and social distancing [8]. Gender stratification of the labor market positions women and girls in lower-wage roles in both formal and informal sectors [9, 10]. Following commercial and school closures, along with restrictions on movement, concern for the economic consequences of the epidemic emerged in Nairobi’s informal settlements, specifically regarding food shortages and income loss [11]. Early evidence revealed a socio-economic gradient, with those at lowest income levels reporting the highest number of contacts during periods of government-imposed restrictions [12]. Subsequent data from youth demonstrate disproportionate repercussions to women in areas of food insecurity, household violence, and forgone healthcare [13].

Youth have received limited attention regarding the gendered impacts of COVID-19 [14], yet this generation will experience long-term economic and social consequences of the pandemic. Simultaneously, gender and gender regimes are uniquely formative for youth [1]. Thus, a gender lens that considers social and economic power disparities is necessary to fully understand the economic disruption of COVID-19—and its corresponding social, health, and safety impacts. Gender is conceptualized as a complex *social system* in which men, women, and gender minorities are differentiated and afforded differential power, resources, and status [1, 15]. Key axes of gender relations include labor (e.g., the distinction of men’s work versus women’s work that gives rise to gendered economic disparities, and workforce stratification [1]) and decisional power (e.g., gender divide in decisional authority, within relationships, the workplace, and governance structures), against the backdrop of social norms and affective attachments that are iteratively shaped by, and simultaneously perpetuate, gender relations [15].

Evidence to date illustrates the profound *economic* impact of business closures and constrained movement caused by COVID-19, which disproportionately affects women [14]. Less is known about the impact on youth, though existing research suggests implications in the domains of health, social support and interaction, and personal safety. Young women’s economic dependence on partners, via transactional sex [16], can be exacerbated during economic disruption. Key *health* indicators include COVID-19 risk perceptions and preventive behavior for which gender differences have been observed [17], as well as health access and disruptions, including those specific to contraception and menstrual hygiene [18]. Youth can experience these disruptions acutely; puberty presents new dimensions of sexual/reproductive health social control and stigma, particularly for women, compromising access to menstrual hygiene supplies and contraception [19–21]. The COVID-19 pandemic has wrought havoc on mental health, though gender disparities are inconsistent across settings [17, 22]. Key *social* indicators include time with partners, social support, freedom of movement, and technology access, given gendered constraints to women’s movement [23] and the digital gender divide [24]. *Safety* concerns are paramount in emergencies [3]; survivor reports from Kenya in the immediate wake of mitigation measures illustrate heightened risk for physical and sexual violence from both partners and strangers, in both homes and public spaces [25]. Reports also describe police violence under the guise of COVID-19 mitigation enforcement [26].

Against this backdrop, we: 1) examine the economic, health, social, and safety impact of COVID-19 on adolescents and young adults in Nairobi, Kenya through mixed methods and 2) characterize the gendered impact by applying gender analysis principles [27], specifically gender contrasts, gender-stratified models, and triangulation of qualitative and quantitative results guided by gender theory that attends to power differentials in social and economic spheres [1, 15]. Results provide timely evidence to guide recovery investments that are responsive to the needs of young men and women.

## Methods

This mixed-methods study draws on cross-sectional survey data collected from August-October 2020 from a cohort of adolescents and young adults; coupled with qualitative focus groups and interviews with youth and stakeholders.

### Quantitative methods

The youth cohort was first recruited in June-August 2019 using respondent-driven sampling (RDS). Eligible youth were age 15–24 years, unmarried, and residing in Nairobi County for at least one year prior to completing the baseline survey. Consistent with RDS methods [28], following an extensive formative phase [29, 30], seeds (n = 9) were purposefully selected to catalyze peer-to-peer recruitment via coupon distribution (up to three per person) until the target sample size was achieved. Following eligibility determination and informed consent, seed participants and subsequent recruits completed survey data collection and received information about support services. In 2019, 1,357 participants were recruited, of whom 95% (1293/1357) provided recontact information and consent. In August-October 2020, 94% (1217/1293) of these participants were recontacted, consented, and completed the follow-up survey. Age, marital status, and Nairobi residence were not eligibility requirements for the follow-up survey.

Due to the COVID-19 pandemic restrictions, data collection was conducted remotely by phone in either English or Swahili, per participant preference. Trained resident enumerators (REs) administered phone-based survey data collection using a dialer and survey programmed in OpenDataKit (ODK). To reduce participant burden, survey content was divided into two separate survey sessions conducted within approximately one week. An oral consent process was conducted with all participants, due to the remote nature of study procedures. Consent was recorded electronically via ODK. The Ethics Review Committee (ERC) at Kenyatta National Hospital/University of Nairobi and the Institutional Review Boards (IRB) at Johns Hopkins Bloomberg School of Public Health waived parental consent for this study and approved use of oral consent processes. All procedures aligned with ethical best practices for sensitive topics [31], including specialized training, privacy protections (auditory privacy screener and protocol), and provision of resource referrals (COVID-19 and violence support). Participants’ compensation (500 KES or US$5 per survey) was transferred via M-Pesa. All procedures were approved by the ERC at Kenyatta National Hospital/University of Nairobi and the IRB at Johns Hopkins Bloomberg School of Public Health.

### Measures

Standard demographic assessments included age, main pre-COVID-19 activity (work [formal sector, informal sector, self-employed], student, other), and subjective household socio-economic status (SES) [32]. Health, economic, social, and safety domains aligned with best practices and leading gender empowerment frameworks. Existing measures were utilized when possible, including from Evidence-based Measures of Empowerment for Research on Gender Equality (EMERGE) [33]. Likert scales were dichotomized based on underlying distributions to maximize statistical power.

#### Health

Three binary COVID-19-related risk and perception items examined the level of concern about community spread of COVID-19 and becoming infected, and participant engagement in at least one prevention behavior. Single items assessed need for healthcare visit, health (among those accessing healthcare), and contraceptive (among contraceptive users) disruptions since COVID-19 restrictions. A single item assessed menstrual hygiene challenges since COVID-19 restrictions, with responses including: could not go to the store; not comfortable asking someone to go to the store; products not available; do not have enough money to buy; other [33]. Depression within the last two weeks was measured via the PHQ2 [34] and dichotomized on probable depression via symptomology (score > = 3).

#### Economic

Inability to meet basic needs since COVID-19 restrictions was assessed using a 5-point Likert scale, ranging from 5-very able to 1-not very able; for outcomes analyses, responses were dichotomized, with 1 = not very able/not able at all and 0 = very able/somewhat able. Household hardship since COVID-19 restrictions [33] was assessed separately by gender, with those living alone coded as “0” for multivariable models. Transactional partnership within the past year was measured for women reporting a past-year partner; additional items assessed timing of onset relative to COVID-19 restrictions and dependence.

#### Social

A 4-point Likert scale assessing current control over decisions to leave the house [33] was dichotomized into full control versus fair/some/little/no control. Among those in partnership, changes to time spent with partner since COVID-19 were characterized. A 5-point Likert scale assessed current social support (can get the emotional help and support I need from people in my life). Frequency of access to safe, private internet [33] was dichotomized (1 = never/rarely; 0 = always/sometimes) and current limitations on access further assessed.

#### Safety

To measure safety at home and in public since COVID-19 restrictions, we used a 4-point Likert scale; for outcomes analysis, safety in public was dichotomized (1 = not safe at all in public; 0 = very safe, somewhat safe, not very safe). Current fear of police harassment, any interaction with police since COVID-19 restrictions, and police demand for money among those in contact were assessed via single items. For young women, past-year experience of intimate partner violence (IPV), non-partner sexual violence, and timing of IPV experiences relative to COVID-19 restrictions were assessed via best practices for violence assessment [33].

### Analysis

All analyses were conducted using Stata 16.1 (College Station, TX). Sampling weights accommodate the RDS study design using RDS-II (Volz-Heckathorn) weights, post-estimation adjustment based on 2014 KDHS population data (age, sex, education levels), and modest adjustment for loss-to-follow-up. All presented estimates are weighted, and statistical testing accounts for clustering among participants recruited by the same recruiter at baseline (node).

Descriptive statistics characterized the study sample overall and by gender. Proportions of key economic, health, social, and safety outcomes were calculated for young men and young women, with significance by gender assessed via design-based F-statistic. When variation in outcome distribution allowed, multivariable logistic regressions used GLM link log and family binomial, and accounted for robust standard error clustering by node and survey design weighting. Models were constructed for outcomes including ability to meet basic needs, need for health care, health care disruptions, contraceptive disruption, depressive symptoms, decisional control to leave the house, internet access, safety in public, and police interaction, and examined gender, as well as a limited set of theoretically relevant, outcome-specific covariates centering on economic and household factors. To evaluate the potential for gender-specific influences on key outcomes, models were then stratified by gender.

### Qualitative study methods

Synchronous online focus group discussions and interviews [35] were conducted via the Zoom videoconferencing platform by trained research assistants. Youth participants (age 15–24 years) and stakeholders at community-based organizations (CBOs) were identified via community-partnered recruitment through the assistance of local youth organizations. Participation in the baseline survey was not a requirement for youth to be selected for the qualitative study. Focus group discussions (FGDs) were conducted in August 2020 with youth (8 FGDs; total n = 64) and youth-serving stakeholders (4 FGDs; n = 32). Following a semi-structured guide, FGD participants were asked about perceptions and youth norms related to family planning, relationships, sexual behaviors, and health, socioeconomic, and safety impacts of COVID-19, including gender dynamics therein. In-depth interviews (IDIs) were conducted with purposefully selected follow-up study cohort members immediately following completion of survey data collection (October 2020). Following a semi-structured guide, IDI participants were asked about personal experiences related to family planning, relationships, and social, economic, and safety impacts of COVID-19, including gender dynamics therein. Oral consent was collected from all qualitative participants, as conducted in the quantitative data collection. Discussions were audio-recorded, transcribed verbatim, and translated to English language (if needed) for inductive and deductive thematic analysis.

## Results

Participants trended towards older ages with secondary level education (Table 1). More young men than young women reported living alone (48.0% vs. 6.3%, respectively, p<0.001). Occupational gender differences prior to COVID-19 restrictions found greater proportions of young men holding jobs and women enrolled as students (p = 0.006).

**Table 1:** Participant demographics, overall and by gender, weighted (n = 1,217).

	Overall	Gender		
		Young men	Young women	p-value
	(n = 1,217)	(n = 605)	(n = 612)	
	**Column %**			
**Age**				0.25
16–18 years	19.4	18.6	20.0	
19–21 years	34.3	31.1	36.4	
22–26 years	46.3	50.3	43.6	
**Highest level of schooling completed**				0.80
Less than secondary	33.8	32.6	34.6	
Secondary / ‘A’ level	56.8	58.3	55.8	
College / University	9.4	9.1	9.6	
**Married/Living with someone as if married**				0.21
No	90.6	92.5	89.3	
Yes	9.4	7.5	10.7	
**Living situation**				**<0.001**
Lives alone	23.4	48.0	6.3	
Lives with parent(s) / parental figure(s) with or without other(s)	54.0	35.9	66.5	
Lives with partner with or without other(s), excluding parents	7.6	4.1	10.0	
Lives with others, excluding parents or partner	15.1	12.0	17.2	
**Main activity before COVID-19 restrictions (January 2020)**				**0.006**
Working	53.6	61.4	48.2	
Student	37.6	33.5	40.5	
Other	8.7	5.1	11.3	
**Subjective Household SES tertile**				0.49
Lowest (1–3)	39.0	41.8	37.0	
Middle (4)	21.7	21.0	22.2	
Highest (5–10)	39.3	37.2	40.7	

### COVID-related risks and perceptions

COVID-19 risk perceptions were high for young adults, with over 80% reporting high concern for community spread and their own risk of infection (community spread: 80.8% young men, 91.7% young women; own infection: 84.2% young men, 95.5% young women; p<0.001; Table 2). All youth had engaged in at least one preventive behavior.

**Table 2:** Key health, economic, social, and safety indicators among Nairobi youth and young adults in August-October 2020; by gender, weighted (n = 1,217).

	Young men	Young women	p-value^†^
	(n = 605)	(n = 612)	
	%	%	
**COVID-19-RELATED RISKS AND PERCEPTIONS**			
Level of concern about the spread of COVID-19 in community			**<0.001**
*Very concerned or concerned*	80.8	91.7	
*A little concerned or not concerned*	19.3	8.3	
Level of concern about becoming infected, among those who did not contract COVID-19 (n = 1,215)^1^			**<0.001**
*Very concerned or concerned*	84.2	95.5	
*A little concerned or not concerned*	15.9	4.5	
Has engaged in at least one preventive behavior against COVID-19^2^	100.0	100.0	--
**ECONOMIC**			
Ability to meet basic needs			0.06
*Very able*	13.8	9.0	
*Somewhat able*	41.2	37.6	
*Not very able/not at all able*	45.0	53.4	
Hardship to the household since COVID-19 restrictions			**<0.001**
*Hardship to male household members*	12.1	5.5	
*Hardship to female household members*	36.6	87.7	
Transactional partnership past year, among those with a past year partner (n = 449 women) ^1^	--	35.6	--
Transactional partnership timing relative to COVID-19, among those involved (n = 160)			
*Before COVID-19 restrictions*	--	15.2	--
*Since COVID-19 restrictions*	--	27.6	
*Both time periods*	--	57.2	
Dependence on transactional partnership since COVID-19, among those involved at both times (n = 106) ^1^			
*Increased*	--	49.1	--
*No change*	--	27.8	
*Decreased*	--	23.1	
**HEALTH**			
Health service disruption			**<0.001**
*Did not attempt to access*	67.8	48.0	
*Attempted to access—no disruption*	12.5	18.6	
*Attempted to access—experienced disruption*	19.7	33.4	
Contraceptive access disruption, among contraceptive users at follow-up (n = 628) ^1^^,^^3^	40.4	34.6	0.29
Menstrual hygiene access challenges (any)*	--	52.0	--
*Challenge*: *Lack of money to buy product*	--	45.0	--
*Challenge*: *Could not go to store to buy*	--	7.8	--
*Challenge*: *Was not comfortable asking someone to go to the store*	--	5.1	--
*Challenge*: *Product unavailable*	--	3.4	--
Depressive symptoms	21.8	24.3	0.50
**SOCIAL**			
Decisional control to leave the house			
*Full control*	53.2	40.0	**0.003**
*A fair amount*	17.4	25.3	
*Some*	14.2	11.9	
*Very little/none*	15.2	22.9	
Changes to time spent with partner, among those currently in a partnership (n = 855) ^1^			0.84
*Increased*	38.5	39.6	
*Unchanged*	15.8	13.8	
*Decreased*	45.7	46.6	
Can get emotional help and support needed			0.19
*Strongly agree*	34.8	37.7	
*Agree*	51.5	47.8	
*Neither agree nor disagree*	11.6	14.0	
*Disagree/strongly disagree*	2.1	0.5	
Has safe and private way to access the internet			**<0.001**
*Always*	40.2	26.1	
*Sometimes*	33.7	39.7	
*Rarely/Never*	26.1	34.2	
Limitations on internet access			
*Cost of data*	67.0	68.8	0.65
*Shares device with others; does not own personal device*	25.7	31.9	0.11
*Lack of privacy*	10.1	11.7	0.53
*Time restrictions enforced by household members*	4.0	6.0	0.29
*Only allowed for work or school purposes*	3.2	6.9	**0.02**
*Other*	1.8	1.1	0.38
*No limitations*	15.5	10.9	0.07
**SAFETY**			
Safety at home since COVID-19 restrictions			0.79
*Very safe*	71.1	70.4	
*Somewhat safe*	20.8	22.7	
*Not very safe/not safe at all*	8.1	6.9	
Safety in public since COVID-19 restrictions			**0.01**
*Very safe*	11.8	5.4	
*Somewhat safe*	22.7	20.3	
*Not very safe*	49.9	52.9	
*Not safe at all*	15.7	21.4	
Interactions with police			
*Fear of police harassment when leaving home*	31.8	27.9	0.30
*Reported any interaction with police since COVID-19 restrictions began*	60.1	38.0	**<0.001**
*Police demands for money or something else among those with contact (n = 576)* ^1^	55.2	43.4	0.06
Experienced violence in past 12 months, by type of violence:			
*Intimate partner violence (IPV) among respondents with partner (n = 449)* ^1^	--	17.3	--
*Non-partner sexual violence*	--	3.0	--
Timing of IPV experiences of relative to COVID-19 (n = 67) ^1^	--		
*Before COVID-19 restrictions only*	--	29.9	--
*Since COVID-19 restrictions only*	--	43.3	--
*Both time periods*	--	26.8	--

^†^Design-based F-statistic.

^1^ Weighted n’s and percentages presented to account for survey design; may not add up to sub-sample raw n’s.

^2^Prevention behaviors: Washing hands with soap and water frequently; Washing hands with hand sanitizer frequently; Avoiding any close contact (2 meters) with people when you; Staying in your home; Wearing something that covers your mouth and nose when you go out; Avoiding shaking hands with others; Coughing/sneezing into your elbow or tissue.

^3^Excludes users of withdrawal or safe days method.

*Responses not mutually exclusive.

Qualitatively, participants were mixed on the perceived level of concern for COVID-19 transmission and infection among youth. Some cited past epidemics like HIV and Ebola, indicating that COVID-19 would resolve similar to previous health crises.

*Honestly, I have not seen anyone around be picked with COVID-19 so it is hard for us to believe. Ebola came and it went. Didn’t HIV come and we got used to it so what is so big with Corona that it will make you instilled with fear throughout?– 19-year-old female FGD participant*

Others expressed greater personal concern for COVID-19, particularly for their older family members.

*Let me say I am 80 percent worried about it because I know it is there. Let me not lie, I am worried about getting it. I am very worried [about my family too]… I fear for their lives. You know my mum is a bit old and my grandma, my grandmother is not very healthy.– 20-year-old female IDI participant*

Lack of concern translated into continued participation in large gatherings, and low uptake of preventative behaviors, including handwashing and mask wearing. Youth noted a number of barriers to engaging in preventative behaviors, including a lack of space to social distance, clean water for handwashing, and money for masks.

*First is lack of water… where I’m coming from there is no supply of enough water… So, you find that regular washing of hands is not easily applicable. Then when it comes to masks, you buy some masks they don’t fulfill that standard that is needed by health. Then, then when it comes to masks too the expenses of buying masks now and then you know, it’s a bit costing.– 24-year-old male IDI participant*

### Economic impact

Approximately half of youth reported being unable to meet their basic needs since the onset of COVID-19 restrictions (45.0% young men; 53.4% young women; Table 2) and young women were more likely to report economic hardship compared to young men (87.7% vs. 36.6%, respectively). One third (35.6%) of young women reported a past-year transactional partnership; for over half (57.2%) the experience was both before and during COVID-19, while 27.6% reported onset since the implementation of COVID-19 restrictions. Among those in transactional partnerships at both timepoints, 49.1% described increased economic dependence on the transactional relationship since COVID-19 restrictions.

An inability to meet basic needs was associated with female gender in multivariable models (adjusted odds ratio [aOR] = 1.21; p<0.05; Table 3). Being in the highest tertile of subjective household SES protected young men and women against the inability to meet their basic needs, compared to being in the lowest tertile for household SES. This pattern of results was observed in both the combined and gender-specific models.

**Table 3:** Multivariable logistic regression of key health, economic, social, and safety indicators, combined and gender-stratified, weighted.

	Full Sample		Gender Stratified Models			
			Young Men		Young Women	
	%	aOR (95% CI) ^±^	%	aOR (95% CI) ^±^	%	aOR (95% CI) ^±^
**ECONOMIC**						
**Inability to meet basic needs**						
Gender—young men	45.0	ref	--	--	--	--
Gender—young women	53.4	**1.21 (1.02, 1.44)** ^*^	--	--	--	--
Living status- with parents	47.4	ref	37.8	ref	51.0	ref
Living status- independent of parents	53.0	1.21 (0.98, 1.50)	49.0	1.42 (0.93, 2.18)	58.2	1.15 (0.89, 1.48)
Pre-COVID activity—income generating	50.8	ref	46.0	ref	55.1	ref
Pre-COVID activity—not income generating	48.9	1.03 (0.86, 1.24)	43.4	1.05 (0.74, 1.48)	51.8	1.03 (0.84, 1.28)
Economic shock to household-hardship to male	51.4	0.99 (0.78, 1.25)	45.1	1.44 (0.90, 2.31)	54.2	1.14 (0.86, 1.52)
Economic shock to household-hardship to female	50.7	1.20 (0.94, 1.53)	42.4	0.92 (0.57, 1.48)	53.1	1.00 (0.76, 1.33)
Household SES—lowest tertile	59.5	ref	52.8	ref	64.7	ref
Household SES—middle tertile	50.3	0.86 (0.70, 1.06)	44.7	0.79 (0.54, 1.17)	53.9	0.87 (0.67, 1.13)
Household SES—highest tertile	40.4	**0.68 (0.56, 0.84)** ^***^	36.4	**0.71 (0.51, 1.00)** ^*^	42.9	**0.66 (0.51, 0.86)** ^**^
**HEALTH**						
**Need for healthcare**						
Gender-male	32.2	ref	--	--	--	--
Gender-female	52.0	**1.59 (1.30, 1.95)** ^***^	--	--	--	--
Ability to meet needs-very	28.9	ref	24.6	ref	33.5	ref
Ability to meet needs—somewhat	45.2	**1.53 (1.05, 2.23)** ^*^	30.6	1.25 (0.71, 2.20)	56.2	**1.68 (0.99, 2.84)** ^*^
Ability to meet needs-unable	46.2	**1.50 (1.04, 2.16)** ^*^	36.0	1.47 (0.84, 2.55)	52.1	1.56 (0.94, 2.59)
**Healthcare disruptions, among those seeking healthcare**						
Gender-male	61.2	ref	--	--	--	--
Gender-female	64.3	1.07 (0.88, 1.31)	--		--	--
Ability to meet needs-very	72.6	ref	58.4	ref	83.7	ref
Ability to meet needs—somewhat	62.1	0.83 (0.62, 1.11)	64.6	1.10 (0.66, 1.86)	61.1	**0.73 (0.55, 0.97)** ^*^
Ability to meet needs-unable	63.0	0.85 (0.64, 1.12)	59.2	1.01 (0.60, 1.71)	64.6	**0.77 (0.60, 1.00)** ^*^
**Contraceptive disruption, among contraceptive users at follow-up**						
Gender-male	40.4	ref	--	--	--	--
Gender-female	34.6	0.82 (0.61, 1.11)	--	--	--	--
Ability to meet needs-very	28.7	ref	21.3	ref	40.3	ref
Ability to meet needs—somewhat	36.2	1.32 (0.76, 2.29)	37.7	1.77 (0.80, 3.93)	35.0	0.87 (0.42, 1.80)
Ability to meet needs-unable	39.5	1.46 (0.85, 2.52)	48.4	**2.28 (1.06, 4.91)** ^*^	33.6	0.83 (0.42, 1.66)
**Depressive symptoms**						
Gender—male	21.8	ref	--	--	--	--
Gender—female	24.3	1.09 (0.80, 1.49)	--	--	--	--
Ability to meet needs-very/somewhat	18.9	ref	21.0	ref	17.2	ref
Ability to meet needs-unable	27.7	**1.46 (1.08, 1.96)** ^*^	22.9	1.09 (0.68, 1.76)	30.5	**1.77 (1.20, 2.63)** ^**^
**SOCIAL**						
**Full decisional control to leave house**						
Gender—male	53.2	ref	--	--	--	--
Gender—female	40.0	0.91 (0.77, 1.08)	--	--	--	--
Ability to meet needs-very	52.4	ref	53.5	ref	51.3	ref
Ability to meet needs—somewhat	44.1	0.95 (0.75, 1.21)	53.9	1.10 (0.81, 1.51)	36.7	0.76 (0.54, 1.08)
Ability to meet needs-unable	44.8	0.90 (0.70, 1.15)	52.4	0.96 (0.69, 1.34)	40.4	0.79 (0.57, 1.09)
Living status- with parents	32.4	ref	35.3	ref	31.3	ref
Living status- independent of parents	60.6	**1.83 (1.50, 2.22)** ^***^	63.2	**1.84 (1.38, 2.44)** ^***^	57.2	**1.82 (1.41, 2.34)** ^***^
**Rare/no private internet access**						
Gender—male	26.1	ref	--	--	--	--
Gender—female	34.2	1.24 (0.96, 1.62)	--	--	--	--
Ability to meet needs-very	22.6	ref	15.9	ref	29.8	ref
Ability to meet needs—somewhat	25.1	1.05 (0.60, 1.84)	18.4	1.19 (0.56, 2.53)	30.3	0.99 (0.50, 1.96)
Ability to meet needs-unable	37.2	1.52 (0.89, 2.59)	36.3	**2.36 (1.15, 4.84)** ^**^	37.6	1.23 (0.64, 2.37)
Job-non-student	27.4	ref	27.2	ref	27.6	ref
Job-student	36.6	**1.31 (1.02, 1.69)** ^*^	24.0	0.84 (0.57, 1.23)	43.9	**1.59 (1.16, 2.17)** ^**^
**SAFETY**						
**Not safe at all in public**						
Gender—male	15.7	ref	--	--	--	--
Gender—female	21.4	1.39 (0.96, 2.01)	--	--	--	--
Ability to meet needs-very	18.4	ref	15.6	ref	21.3	ref
Ability to meet needs—somewhat	14.2	0.78 (0.40, 1.51)	10.1	0.68 (0.26, 1.75)	17.4	0.83 (0.33, 2.08)
Ability to meet needs-unable	23.0	1.21 (0.65, 2.25)	20.8	1.36 (0.60, 3.07)	24.3	1.14 (0.47, 2.74)
Living status- with parents	18.2	ref	12.7	ref	20.3	ref
Living status- independent of parents	20.1	1.17 (0.83, 1.65)	17.3	1.24 (0.66, 2.31)	23.7	1.14 (0.74, 1.74)
**Any police interaction**						
Gender—male	60.1	ref	--	--	--	--
Gender—female	38.0	**0.72 (0.60, 0.85)** ^***^	--	--	--	--
Ability to meet needs-very	55.3	ref	64.1	ref	45.9	ref
Ability to meet needs—somewhat	38.8	0.79 (0.63, 1.00)	50.6	0.84 (0.65, 1.10)	29.9	0.69 (0.44, 1.09)
Ability to meet needs-unable	51.6	1.02 (0.83, 1.24)	67.5	1.04 (0.82, 1.31)	42.4	0.94 (0.63, 1.42)
Living status- with parents	34.8	ref	41.8	ref	32.1	ref
Living status- independent of parents	61.4	**1.56 (1.30, 1.86)** ^***^	70.3	**1.62 (1.27, 2.08)** ^***^	49.7	**1.50 (1.15, 1.94)** ^**^

GLM models with link log and family binomial, accounting for robust standard error clustering by node and survey design weighting.

*p<0.05

**p<0.01

***p<0.001.

aOR = adjusted odds ratio.

^±^ adjusted models include all covariates presented.

While survey data lack a pre-pandemic baseline on unmet economic needs, qualitatively, young men and women described profound economic impact via job loss and loss of income for themselves and their families in the wake of the COVID-19 restrictions, prompting financial uncertainty, loan-taking, and food insecurity.

*[Lack of] house rent has seen my mum and my grandmother almost being thrown out of the house.‬.. It is my grandmother who begs [the landlord]. She tries to talk to them in a nice voice and tell them it is just because of the Corona times.‬.. He cannot afford to throw us out because he is a human by the end of the day. Another financial challenge is about the food but I still thank God at least we can eat once in a day… there are some people who usually go without food.– 20-year-old female IDI participant*

*You find that a member of the family maybe used to work in a certain industry… then due to this COVID-19 they lose their jobs… So the breadwinner becomes one person [instead of having two] so challenges are many in the house… Shopping for food for the house, everything has just changed… You may find even that in some families both the breadwinners have lost their jobs.– 15-year-old male FGD participant*

*I have seen after people losing their jobs mostly there is a design they have started hustling. You find that even though children are not at school there is a design they have started to look for themselves money. They themselves are selling fruits on the road. - 19-year-old female FGD participant*

Young women specifically perceived financial constraints as a barrier to resources (i.e., data bundles, stable internet, textbooks) for continued education during COVID-19.

*You find some other… cannot access internet. So their child cannot go to study online classes. You find another one the parent cannot buy the textbooks, because like also meals to them is a problem, they cannot buy milk… if they came from deep inside Ghetto, the mother when she comes she only have one hundred which is enough to buy food only. It cannot be enough to buy books.‬‬‬‬ – 22-year-old female FGD participant*

### Health

As compared with young men, significantly greater proportions of young women attempted to access health services during COVID-19 and experienced disruptions to access (19.7% young men; 33.4% young women; p<0.001; Table 2). Approximately 40.4% of young men and 34.6% of young women using contraception faced difficulty procuring their method(s). Over half of young women (52.0%) reported a challenge procuring menstrual hygiene products, overwhelmingly due to a lack of money (45.0%). Symptoms consistent with depression were comparable between genders and reported by 21.8% of young men and 24.3% of young women.

Young women’s elevated healthcare needs relative to young men’s remained significant after accounting for ability to meet basic needs (aOR = 1.59; p<0.001; Table 3). In gender-stratified models, young women faced an increased need for healthcare as their ability to meet basic needs decreased (aOR = 1.68; p<0.05); conversely, health disruptions for young women decreased with reduced ability to meet needs (aOR = 0.77; p<0.05). Young men unable to meet their basic needs disproportionately faced contraceptive disruptions (aOR = 2.28; p<0.05). Depressive symptoms were associated with an inability to meet basic needs in the combined gender model (aOR = 1.46; p<0.05) and young women’s model (aOR = 1.77; p<0.01).

Qualitative data corroborated that COVID-19 barriers to health centered on fear of accessing services, financial barriers, and facility closures. All groups highlighted COVID-19-related fear as the predominant barrier to accessing hospital and contraceptive services.

*So, going to hospitals where actually people leave the Coronavirus, they are taken and admitted, and the doctors are trying to help them is a major risk. We are exposing ourselves. We are afraid to go because we might get it.– 15-year-old male FGD participant*

*You cannot go to the hospital. There could be rumors that you are disappearing at night and then you get sick, with that rumor you start to sneeze, cough and most of them say it is COVID. So, you cannot even go to the hospital. So, this COVID has affected so much that.– 17-year-old female FGD participant*

With limited financial resources, young people prioritized basic needs, like rent and food, above contraception and sanitary pads.

*Coronavirus has affected the economic status of people… for example, people who don’t usually like going to public hospitals to pick these contraceptives they now go to chemists in private hospitals to pick them and this is money… Something like that people don’t have money for… somebody choses between contraceptives and whatever, food, which will he/she buy?– 21-year-old female FGD participant*

Some youth discussed closure of services and contraception distribution sites, which particularly affected condom users.

*With young men, before COVID started, in the hospital there was this place for keeping condoms. Right now, they don’t keep it. So that they can try to avoid that overcrowding or those people who keep coming to hospital every time.– 22-year-old female FGD participant*

Youth described mental health consequences of the COVID-19 pandemic. Feelings of depression and stress among youth were attributed to job loss and financial constraints, compounded by idleness in the home.

*I think due to lack of job opportunities and the way people have lost their jobs, man, it has led to depression. You are there and there are people who want to eat, I mean they depend on you… I think most of the people are getting depressed… I mean, till you are losing it, you are really losing it, you don’t know what you will do.– 19-year-old female FGD participant*

Both stakeholders and youth pointed to isolation as a chief concern, and highlighted needs for youth-oriented mental health services and counseling.

*I’ve missed some of my friends… then also keeping away people that, that you love, you know. It’s not; it’s not easy. Aah for the last two months, I was not that able much because I was mostly using the social media, yeah. And you know the expenses of social media so that could not allow me to always contact them regularly. 24-year-old male IDI participant*

*At the moment the greatest need according to me for youth now is psychological support. … the way conditions are now considering COVID-19, the way status of the family is, you see how status has been, the way poverty has started to reign in the family…. So, [youth] see as if there is no other solutions on the problems they have now. So right now they need counseling [on] how they will handle these stress they have at the moment—15-19-year-old male FGD participant*

### Social

During COVID-19, control to leave the house differed significantly by gender (p = 0.003; Table 2), with women indicating less control over their decision. Almost half of both young men and young women reported decreased time spent with their partner as a result of COVID-19 restrictions, though just over a third reported increased time with partners. Over three-quarters indicated high social support and strongly agreed or agreed that they were able to get needed emotional help and support. Inability to access safe and private internet disproportionately impacted young women (26.1% young men; 34.2% young women; p<0.001).

Across combined and gender-stratified multivariable models, living independent of parents was associated with increased decisional control to leave the house (p<0.001; Table 3). Students were less likely to be able to access private internet than non-students in combined (aOR = 1.31; p<0.05) and woman-specific (aOR = 1.59; p<0.01) models. In male-only models, young men with lowest abilities to meet basic needs indicated increased inability to access private internet (aOR = 2.36; p<0.01).

Qualitative data highlighted unique privacy constraints given COVID-19 mitigation measures that prompted more time at home. The economic disruption of COVID-19 led families to move into smaller homes with extended family members, limiting privacy. Young women described unique privacy constraints including basic hygiene, getting dressed, and studying in the home.

*At the moment, there is no one going to work outside of the house… So, everyone is in the house, both parents. Covid 19 has led to loss of jobs… So it forces parents to move to smaller houses. Of which it will affect the privacy of everyone… You find that there your relatives have been chased away because of rent… They come and stay with you in the house… So, you see if you had your own room, you have to share.– 19-year-old male FGD participant*

*Sometimes you want to dress… maybe it is a single room… they [male family members] come and go… you want to dress up now you are stuck. How will you dress and those men are there? You say there is no privacy for you as a girl.– 23-year-old female FGD participant*

Young women described constrained autonomy and mobility, including needing parental permission or supervision from older, male relatives to leave the house. In comparison, mobility barriers for young men focused on fear of contracting COVID-19 and of police harassment.

*Right now… you must tell your parents where you went to, even when you just want to leave, they tell you if you have got brothers… They will go with you wherever you go. So, even when you want to go to the shop you are told to go with your elder brother.– 17-year-old female FGD participant*

*[Before COVID-19] I could go to different places. I could go any time wherever I want because I always, I was always on my own principles… but… I fear that, that maybe I will go to somewhere then maybe I can contract the Covid.– 24-year-old male IDI participant*

Stakeholders explained mobile technology as a lifeline for youth to remain connected with social networks; yet the scarcity of privacy undercut this tool, prompting some young women to seek privacy outside the home, even in restrooms.

*The young people most of them they like [to be]… social with their friends, with their peers, so they are now… being restricted to stay at home or even if they go, there is no social gathering. So that safe space for them, there is no safe space for the young people, be it at home [or] be it outside… Now the technology is moving whereby… you can… talk through WhatsApp… or Facebook chat. But in the household level… all people are within [the home,] you are not be able even to talk privately with that person.– 35-40-year-old male youth advocate*

*[To have private communication you go] In paid toilets… Or you go down there to the river… In a place you are not known, you talk about your stories and then come back.– 24-year-old female IDI participant*

### Safety

During COVID-19, both young men and young women felt moderately safe at home (>70%) during COVID-19 restrictions (Table 2). Young women disproportionately felt unsafe in public spaces (15.7% young men; 21.4% young women; p = 0.01). Interactions with police since COVID-19 restrictions were more prevalent among young men compared to young women (60.1% vs. 38.0%, respectively; p<0.001); 55.2% of young men’s contacts included police extortion (money or bribes). Female gender was protective against police interaction in multivariable models (aOR = 0.72; p<0.001; Table 3). Living independent of parents significantly increased odds of police interaction for combined and gender-stratified models (p<0.001). Nearly one in five young women with a past-year partner reported past-year IPV (17.3%), with almost half of IPV experiences occurring for the first time after COVID-19 restrictions were imposed (43.3%).

Qualitative data revealed that social restrictions of COVID-19 prompted new risks for violence, with an emphasis on partner and sexual violence for young women and police violence for men. Specifically, youth perceived curfew restrictions as posing risk for partner violence through the inability to leave a partner’s home after curfew.

*According to me, like now there are curfew hours so maybe you went out and time really went by without you noticing and you remained there, and your parents didn’t know where you went. And now, you are with this guy…And now he has the chance to do with you anything he likes because he knows you can’t go anywhere, it is past curfew, you can’t leave the house. So, he might do anything to you.– 17-year-old female FGD participant*

For others, COVID-19 restrictions offered an excuse to leave a harmful relationship.

*According to me it has affected positively. Because before, the violence was there so much but nowadays it is hard to meet, and when you meet it is hard. So, if there is any violence which comes up, you break up. –18-year-old female FGD participant*

Some young women and stakeholders also discussed concerns for violence within the home perpetrated by family members: “Most people are idle; people lost their jobs. Their uncles, their whatever, now they are coming to prey on young girls and they don’t even have people to support them” (17-year-old female FGD participant).

Conversely, young men’s safety concerns centered around community and police violence, particularly in relation to curfews.

*I think young people are being violated based on this Corona virus because*, *let me say for example*, *you are supposed to be at home by 9*, *and by mistake you find yourself outside and if you might meet police and they might brutally beat you*, *or beat you physically*, *which is abusive*. *-18-year-old male FGD participant*

Triangulation of qualitative and quantitative results (Table 4) illustrates areas of convergence and divergence, and synthesizes gendered impact across data sources.

**Table 4:** Triangulation of quantitative and qualitative results, synthesis and gender analysis.

Quantitative Key Results	Qualitative Key Results	Synthesis and Gender Analysis
**COVID-19-RELATED RISKS AND PERCEPTIONS**		
• COVID-19 related concern and risk perception is high overall, modest gender difference favors young women (concern: 91.7% young women, 80.8% young men; risk perception: 95.5% young women, 84.2% young men) • All youth reported having engaged in at least one preventative behavior	• Youth were mixed on their level of concern for COVID-19 transmission • Significant barriers discussed to preventative health behaviors, including social and financial barriers	• Divergence between quantitative and qualitative, with description of mixed concern for transmission and difficulty practicing preventative behaviors • Modest gender difference favoring young women on COVID-19 concern and risk perception
**ECONOMIC**		
• Widespread inability to meet basic needs (45.0% young men; 53.4% young women) • Young women disproportionately unable to meet basic needs (aOR = 1.21; p<0.05), even accounting for buffering effect of household economic status • 35.6% of young women with a past year partner engaged in transactional partnership; greater dependence (49.1%) for those involved both pre- and during COVID-19	• Severe economic impact to both young men and young women due to job loss and loss of income • Repercussions include financial uncertainty, loan-taking, and food insecurity • Informal labor opportunities are limited for young women • Young men increase reliance on criminal activities, including robbery and digital scams, to generate income	• Universal economic impact to youth consistent across qualitative and quantitative • Disproportionate economic impact on young women • Gender specific: young women’s dependence on transactional partnerships increased
**HEALTH**		
• Young women more likely to attempt to access health services, compared to young men (aOR = 1.59; p<0.001) • 34.6% of young women and 40.4% of young men using contraception had trouble accessing their method • Gaps in access to menstrual hygiene products for young women (52.0%), with lack of money the most common challenge (45.0%) • Depressive symptoms for young men (21.8%) and women (24.3%)	• Fear discussed as most prominent barrier to accessing health services, including contraception • COVID restrictions force closures on services that youth previously used to access contraception, particularly coital-dependent methods • Financial barriers for contraceptive access and menstrual hygiene • Stress due to financial hardship, job loss, disruptions to academic plans, and idle time due to COVID-19 restrictions contribute to poor mental health	• Gender difference in health service disruption, with young women more likely to need care • Fear of infection was the dominant barrier to accessing healthcare • Contraceptive disruptions common, particularly for coital-dependent methods • Gender specific: Menstrual hygiene barriers are common and primarily financial • Gender symmetry: Mental health impact of COVID-19
**SOCIAL**		
• 22.9% of young women and 15.2% of young men reported very little control to leave the house • Only a quarter (26.1%) of young women report having safe, private access to internet. • The greatest limitation on internet access was cost of data for both young men and young women • Nearly half of young men (45.7%) and women (46.6%) are spending less time with their partners • High perceived social support for both young men and young women	• Increased time at home to youth and their family members • Young women face greater household privacy constraints, and are more limited in mobility compared to young men • Differentiated gender barriers: permissions/supervision for women, fear of police harassment and COVID-19 among men • Young men report constraints to mobility primarily around fear of contracting COVID-19 and fear of police harassment • Technology and mobile phones enable connectivity, yet privacy barriers can undermine access	• Young women face greater privacy and mobility challenges • Full decisional control to leave house favors young men bivariately, and youth no longer living with their families • Gendered gaps in technology, with young women less likely to have safe private internet access bivariately, and students disproportionately impacted • Gender symmetry: mixed impact on time with partners • Gender symmetry: high social support
**SAFETY**		
• Safety concerns centered outside of the household; relative safety within the home for over 70% of young men and young women • Police interactions since COVID-19 disproportionately affected young men (aOR = 0.72; p<0.001), with over half of their interactions involving extortion • Nearly 1/5 of young women experienced past-year IPV, with (70.1%) occurring since COVID or both timepoints	• Police violence and harassment related to COVID-19 curfew and restrictions • COVID-19 restrictions create risk of gender-based violence for young women; in some cases, COVID-19 provides a cover for relationship breakup following safety concerns • Young people face risk of sexual violence from male family members before and since COVID-19	• Gender differentiated safety risks: • young men face police violence/harassment • young women face sustained risk for partner violence, both pre and during COVID-19; non-partner sexual violence was less prevalent

## Discussion

Findings from this mixed-methods study demonstrate significant unmet economic, health, social, and safety needs for youth and young adults in Nairobi during the COVID-19 pandemic. Concerning gender disparities emerged with young women more economically vulnerable and in greater need of healthcare, while young men faced heightened risk for police violence relative to young women. Gender-specific risks include young women’s sustained risk for IPV, increased reliance on transactional partnerships, and gaps in access to menstrual hygiene products. Gender symmetry was identified in mental health burden, and, encouragingly, in the social support that youth felt despite the hardships. Inclusion of a comprehensive set of indicators reflecting both gender and developmental considerations enables understanding of COVID-19’s impact on a population that will long grapple with its disruption. While underlying disparities versus COVID-related influences cannot be fully assessed quantitatively, qualitative results speak clearly to the pandemic’s impact on economic security, safety, mental health, and access to essential sexual/reproductive health services. The COVID-19 pandemic appears to have amplified existing gender disparities for youth and young adults in Nairobi, and risks disproportionate threats to young women’s health and livelihoods. Pandemic recovery efforts for youth must take a gendered lens, and work to overcome the normative, social, and economic systems that give rise to underlying gender disparities.

The economic distress for youth and their families was universally expressed, and an alarming 45% of young men and 53% of young women were unable to meet their basic needs at the time of the survey, echoing reports inclusive of a wider age range [36]. Even with near-universal impact, economic hardship disproportionately affected young women in adjusted models (aOR = 1.21), even after accounting for the buffering effect of socio-economic status. Women’s increased reliance on transactional partnerships since the mitigation measures (49% among those involved) further illustrates the confluence of gendered economic and social dynamics. Together, findings illustrate that economic hardship of COVID-19 is not felt equally where gender stratification of the workforce exists alongside entrenched gendered socioeconomic disparities. Results echo and extend a global evidence base that overwhelmingly points to the gendered economic impact of COVID-19 [14], by focusing on youth who are transitioning from education into the labor force and risk long-lasting impacts of economic setbacks and disparities. Economic and social investments, including cash transfers, must be rapidly implemented to lift the immediate financial crisis to individuals and families, with an emphasis on those most vulnerable. Other solutions include prohibitions against eviction during the crisis, and increases in access to essential and basic health supplies and services through local clinics and pharmacies.

The economic fallout of the pandemic contributed to a cascade impact on youth health, social, and safety outcomes, particularly among young women. Inability to meet basic needs was a strong predictor of depressive symptoms in the women’s stratified model, suggesting intersectional gender and economic influences. Among the 52% of young women who reported barriers to menstrual hygiene products during the pandemic, financial gaps were the primary driver rather than mobility restrictions or supply disruptions. Socio-economic disparities in access to menstrual hygiene products have been documented pre-pandemic [37]; current data are imprecise as to the pandemic-specific influences. Nevertheless, inclusion of free menstrual hygiene products within mitigation efforts is a concrete step to lift this gendered burden.

Consistent with earlier reports from Nairobi [13], health disparities disproportionately affect young women, reflecting their relatively higher needs for health care during this time (aOR = 1.59). Global concern has emerged for contraceptive interruptions and resulting unintended pregnancy [38]. Despite the relative gender symmetry in contraceptive disruption, the social and health impact of contraceptive gaps and resulting unintended pregnancy falls disproportionately to women, particularly when they are young and unmarried. Our study’s short follow up period did not permit exploration of resulting unintended pregnancy in this wave of data collection, though future analysis should explore the longer-term impact of contraceptive disruptions on pregnancy, and educational and economic attainment. Expansion of access points for free condoms and other contraceptive methods, and menstrual hygiene supplies, can extend to free, discreet distribution at local pharmacies, health clinics, markets, and COVID-19 response sites to ensure safe access for youth through the mitigation measures of the pandemic. Youth-friendly, clear, and accurate public health messaging is needed to address fears concerning COVID-19 acquisition in medical facilities, and support youth in meeting their health needs while mitigating risk. These interventions require social and policy recognition that sexual and reproductive health care is an essential health service, including among unmarried youth.

Study findings shed new light on gendered social dynamics among youth during public health emergencies. Mobility restrictions disproportionately impacted young women, who faced gaps in decisional control to leave the house. Qualitative evidence illustrated gendered requirements for permission and supervision. Similar gender-based constraints to the outside world during pandemic restrictions have been observed among youth in Jordan [39], echoing gendered restrictive social norms that can curtail women’s freedom of movement [23], and demonstrate the persistence of mobility disparities even in public health emergencies with widespread mobility restrictions. Current evidence reveals a mixed impact on time with partners; for some youth the restrictions may curtail opportunities to connect with partners, while for others, the lifting of school or work responsibilities may enable more time with partners.

Gender gaps were also revealed in safe and private internet access, raising concerns about the ability of mobile technology to fulfill needs spanning remote learning, social connection, entrepreneurship, and discreet access to health and social services during the COVID-19 pandemic. Young women were at significantly greater risk than young men for having no or rare private safe internet access; among women, risks were greatest for those in school pre-pandemic. Findings echo concern for the digital gender divide [24] and highlight sustained needs for young women’s access and ability to benefit from mobile technology.

Safety concerns for both young men (15.7%) and women (21.4%) emphasized risks in public spaces, with profound gender differences in the nature of safety threats. Echoing human rights reporting [26], 60% of young men reported police interactions since COVID-19 restrictions, of which over half were police demands for money/bribes (55.2%). Police interactions were concentrated among young men, who qualitatively expressed fear of police brutality. This gender disparity may reflect gender differences in mobility, and women’s greater likelihood of staying home. Young women’s safety risks centered on IPV; in this sample of primarily non-cohabitating youth, 17% of young women with a past-year partner report past-year IPV. Among those affected, timing relative to COVID-19 was mixed, with 43% reporting pandemic-related onset, and 27% experiencing IPV both prior to and during COVID-19. Results may reflect financial stress and increase in conflict over meeting basic needs in the context of scare resources. While the global dialogue has centered on adults and cohabitating couples for IPV-related pandemic impact, current results highlight the need to address IPV among youth during public health emergencies. In contrast, despite concerns for sexual violence by family members and strangers, the past-year prevalence of non-partner violence in this population was 3%.

Several limitations should be considered. Despite the extensive interviewer training and privacy protections in place for phone interviews, errors, underreporting, and social desirability biases are possible, particularly for more sensitive items such as sexual violence. Despite the mobile connectivity of Nairobi, results from this phone-based survey may not fully represent the underlying population of young people, particularly those for whom phone access and use are intermittent. Survey data collection was conducted during a relative low point in COVID-19 case load in Kenya; perceptions and experiences may evolve rapidly through the course of the pandemic. We lacked pre-COVID assessments for most indicators, thus we are unable to quantify pandemic-specific gender impact relative to underlying gender disparities and economic disparities that predated the pandemic; qualitative data aid in understanding the COVID-19 related influences. Though IDI participants discussed their first-hand experiences related to contraception and COVID-19, FGD participants were asked about personal perceptions and youth norms (as is conducive to group discussions) related to these topics. Thus, some qualitative data from FGDs may not be representative of actual youth experiences. Overall, FGD findings nonetheless converged with key themes from the IDIs.

The COVID-19 pandemic has had profound implications for youth and young adults in Nairobi. Current evidence of gender disparities and gender-specific risk in Nairobi likely reflect a combination of pandemic-specific influences together with underlying disparities. Results suggest the potential for far-reaching social, economic, and health consequences for generations to come. As youth transition back to school, monitoring is needed to ensure gender equity and offset potential gaps, including those related to supplies, school fees, and technology access. Global and national COVID-19 recovery represents an opportunity to rectify gender and socio-economic disparities while mitigating the pandemic’s disruption. Gender-inclusive and youth-inclusive decision-making are essential, yet concerning gender gaps persist in COVID-19 decision-making entities [40], including in Kenya. Gender-responsive guidance [40] must shape response and recovery efforts, including meeting gender- and age-differentiated needs, and ensuring gender-stratified monitoring and impact evaluation. The COVID-19 pandemic represents a global wake-up call on gender and socio-economic disparities; equity must drive its resolution. To effectively respond and potentially limit the long-term economic, health, education, and safety impacts for young men and women, governments must have the courage to make bold and comprehensive investments in their populations, with a targeted and gender responsive approach to meet the needs of those most impacted by the pandemic.
